# A new score including CD43 and CD180: Increased diagnostic value for atypical chronic lymphocytic leukemia

**DOI:** 10.1002/cam4.3983

**Published:** 2021-06-01

**Authors:** Yi Li, Xiwen Tong, Lifang Huang, Li Li, Chunyan Wang, Cheng He, Songya Liu, Zhiqiong Wang, Min Xiao, Xia Mao, Donghua Zhang

**Affiliations:** ^1^ Department of Hematology Tongji Hospital Tongji Medical College Huazhong University of Science and Technology Wuhan China

**Keywords:** atypical, chronic lymphocytic leukemia, flow cytometry, immunophenotype

## Abstract

Moreau score has been used to differentiate chronic lymphocytic leukemia (CLL) from other mature B‐cell neoplasms. However, it showed limitations in Asian patients. Therefore, we conducted a new score system replacing CD5 and CD23 with CD43 and CD180 to evaluate its diagnostic value of CLL. 237 untreated samples diagnosed with mature B‐cell neoplasms were collected and were randomly divided into an exploratory and a validation cohort by a 2:1 ratio. The expression of CD5, CD19, CD20, CD23, CD43, CD79b, CD180, CD200, FMC7, and surface immunoglobulin (SmIg) were analyzed among all the samples. A proposed score was developed based on the logistic regression model. The sensitivity and specificity of the proposed score were calculated by ROC curves. CD43/CD180, CD200, FMC7, and CD79b were included in our new CLL score, which showed a sensitivity of 91.8% and a specificity of 83.1%. These results were confirmed in a validation cohort with a sensitivity of 90.5% (*p* = 0.808) and a specificity of 79.5% (*p* = 0.639). In CD5 negative or CD23 negative CLL group, the new CLL score displayed improved sensitivity of 79.4% compared to Moreau score and CLLflow score (41.2% and 47.1%, respectively). In atypical CLL group, the new CLL score showed improved sensitivity of 84.2% compared to Moreau score and CLLflow score (61.4% and 64.9%, respectively). This proposed atypical CLL score helped to offer an accurate differentiation of CLL from non‐CLL together with morphological and molecular methods, particularly in Chinese patients with atypical immunophenotype.

## Graphical abstract

This study demonstrated the diagnostic value of combination of CD43 and CD180 with CD200, FMC7 and CD79b in CD5‐ or CD23‐ CLL for the first time. How to differentiate atypical CLL from other mature B cell neoplasms poses a problem in the diagnosis of CLL. This study investigated the immunophenotyping data of patients with mature B‐cell malignancies, and developed a new combined score including CD43 and CD180 which could improve the diagnostic value of CLL versus non‐CLL, particularly in atypical CLL.

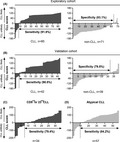

## 1 INTRODUCTION

Chronic lymphocytic leukemia (CLL) is a clonal disorder characterized by expansion of mature‐appearing B lymphocytes in the blood, bone marrow, lymph nodes, and spleen.^1^ The diagnosis of CLL requires the persistent presence of monoclonal B cells ≥5 × 10^9^/L in the peripheral blood with characteristic immunophenotypes.^2^, ^3^ CLL typically expresses CD5, CD23, bright CD43, dim‐to‐negative CD79b, CD200, and dim surface immunoglobulin (SmIg).^2^, ^4^ Flow cytometry is a well‐established tool in the evaluation of peripheral blood, bone marrow, and disaggregated tissue biopsies,^5^, ^6^ and is vital for the diagnosis of typical CLL with great precision and accuracy.^7^

A scoring system for the diagnosis of CLL was first defined by Matutes et al^8^ consisted of CD5 and CD23 positivity, FMC7 negative as well as CD22 weak or negative and SmIg weak. CD22 was replaced by CD79b in Moreau score^9^ and a score of 4–5 indicates typical CLL and a score of 3 or less indicates atypical CLL or excludes CLL.^8^, ^9^ In Asian populations, however, atypical immunophenotypic features were more common,^10^, ^11^, ^12^ and were difficult to be diagnosed precisely according to Matutes or Moreau score.^13^, ^14^ Recently, a revision called CLLflow score, based on the immunophenotype: (%CD200 positive) + (%CD23/CD5 positive)—(%CD79b positive)—(%FMC7 positive), predicted CLL with a score greater than zero.^15^ This score demonstrated improved specificity (87.2% vs. 53.8%) compared with Moreau score.^15^ However, 7%–20% of CLL patients are negative for CD5,^16^, ^17^ and 16% of CLL patients were CD23 dim,^18^ which may lead to decreased sensitivity as CD5 and CD23 were the positive markers in both Moreau and CLLflow score. Therefore, new markers with higher positive expression on CLL are required for improving the diagnostic value.

CD43 is a surface molecule expressed on T cells but positive in CLL.^19^ CD43 positivity was a marker with a sensitivity of 100% for CLL diagnosis.^20^ CD 43 is recommended for diagnosing CLL in borderline cases by the International Working Group CLL (iwCLL)^21^ and European consensus.^22^ CD180 is a toll‐like receptor homolog protein expressed on B cells.^23^, ^24^ The differential expression of CD180 among chronic B‐cells lymphoproliferative diseases has been reported.^24^, ^25^, ^26^ This study aimed to investigate the immunophenotyping data of patients with mature B‐cell malignancies, and whether a new combined score including CD43 and CD180 could improve the diagnostic value of CLL versus non‐CLL, particularly in CLL with CD5 or CD23 negativity.

## 2 MATERIALS AND METHODS

### 2.1 Study population

Flow cytometric results from patients who were newly diagnosed with mature B‐cell neoplasms from October 2015 to October 2019, were reviewed retrospectively. The diagnosis were made according to the World Health Organization (WHO) 2008 classification^27^ and WHO 2016 classification.^28^ CLL cases with atypical immunophenotype including Moreau score ≤3, and CD5 or CD23 negativity were prudently diagnosed based on clinical features, cytomorphology and molecular genetics with absence of t(11;14)(q13;q32). The inclusion criteria were (a) patients who were evaluated for CD5, CD19, CD20, CD23, CD43, CD79b, CD180, CD200, FMC7, and SmIg expression using flow cytometry and (b) the initial diagnosis was first established by experienced physicians taking comprehensive account of clinical, cytomorphological, immunophenotypic, molecular, cytogenetic (and/or fluorescent in situ hybridization [FISH]) characteristics and immunohistochemistry, if available. Then the therapeutic efficiencies and prognosis of all patients were dynamically analyzed, and the initial diagnosis of CLL or non‐CLL were finally confirmed in a follow up. Exclusion criterion was previous presence of hematological malignancies other than mature B‐cell neoplasms. A clinical chart of patient selection was displayed in Figure S1. The present study was approved by the Ethical Committee of our Hospital, and all procedures conducted were in agreement with the Declaration of Helsinki.

All the cases were evaluated according to the Moreau score (CD5, CD23, FMC7, CD79b and sIg)^9^ and CLLflow score (CD5/CD23, FMC7, CD79b, and CD200).^15^ Then, CLL patients with Moreau score ≤3 were identified as atypical CLL group and CLL patients negative for CD5 or CD23 were selected as CD5 negative or CD23 negative CLL group. Samples evaluated by flow cytometry were consisted of bone marrow aspirations, lymph node biopsies, peripheral blood and spleen, with no difference in the scores among different specimens.

### 2.2 Flow cytometry

All the patient samples obtained were subjected to flow cytometric examination, and 2 × 10^6^ cells/tube were incubated and lysed manually using an erythrocyte lysing reagent (Uti‐Lyse, Dako) for 8 min. The cells were then washed in phosphate‐buffered saline/10% fetal bovine serum and re‐suspended in phosphate‐buffered saline for acquisition. Lastly, the samples were stained with fluorochrome‐conjugated monoclonal antibodies at room temperature, for 15 min in the dark. The major antibodies used were listed in Table S1.

Lymphocytes were selected by gating on CD45high/side scatter (SSC) low events. B‐cell population from all suspicious B‐cell neoplasms was selected as CD19 positive or CD20 positive (CD20 was used when CD19 showed weak expression). Immunophenotypes were detected using multiparameter flow cytometry (LSRFortessa, Becton Dickinson) with acquisition target set at 1,000,000 leukocyte events. Data were analyzed using BD Diva software. A marker was considered positive when expressed over 20% of the B cells^15^ and the expression of a marker was represented according to published recommendation.^29^ The expression of a marker was classified as “negative” (≤20%), “part expression” (>20%–80%) and “expression” (>80%) assessed according to the positive rate. The strength of antigen expression was measured depending on the mean fluorescence intensity (MFI) and the ratio of fluorescence intensity (RFI) corresponding to the MFI normalized to the MFI of the isotype negative controls.

### 2.3 Statistical analysis

Continuous variables were compared using independent group t‐tests or one‐way ANOVA for normally distributed data. Data were analyzed by the Mann‐Whitney U‐test or Kruskal‐Wallis H‐test when the data were not distributed normally. Categorical data were compared using *χ* ^2^ test, or Fisher's exact test when the data were limited. Receiver‐operator characteristic (ROC) curves analysis and Youden index of markers were performed to determine the maximum likelihood value that could distinguish CLL from non‐CLL. Logistic regression model (entering) was performed to determine the diagnostic value of all candidate markers in a combined model. The collinearity diagnosis was performed to evaluate the relationships among variables in the model. The goodness‐of‐fit of the model to the data was evaluated by calculating the Hosmer–Lemeshow statistics. The proposed score was constructed based on the logistic regression model in the exploratory cohort. The proposed score construction and validation were based on published articles.^15^, ^30^ Finally, the sensitivity and specificity of the proposed score in exploratory cohort and validation cohort were compared using *χ* ^2^ test. The comparison between the proposed score and Moreau score or CLLflow score were used McNemar's test. Analyses were performed with SPSS, version 24.0 (IBM Corp.). Statistical significance was considered if *p* < 0.05.

## 3 RESULTS

### 3.1 Patient characteristics

A total of 555 samples from untreated patients with mature B‐cell neoplasms were analyzed by multiparameter flow cytometry. Finally, 237 samples of them who met all criteria were included in this study. There were 127 CLL cases and 110 non‐CLL cases. Non‐CLL cases included diffuse large B‐cell lymphoma (DLBCL), Burkitt lymphoma, mantle cell lymphoma (MCL), follicular lymphoma (FL), marginal‐zone lymphoma (MZL), extranodal marginal‐zone lymphoma of mucosa‐associated lymphoid tissue (MALT), and lymphoplasmacytic lymphoma (LPL) (Table S2). Patient cohort was randomly divided into an exploratory and a validation cohort by a 2:1 ratio (Table S2). Information regarding clinical characteristics, molecular genetics and cytogenetics of CLL patients in exploratory cohort and validation cohort is shown in Table S3. The comparison of clinical characteristics between CLL patients with CD5 negativity and CD5 positivity is listed in Table S4.

### 3.2 Positive rate (> 20%) of CD43 and CD180 expression in CLL and non‐CLL patients

#### 3.2.1 The CD43 expression between CLL and non‐CLL patients

**FIGURE 1:**
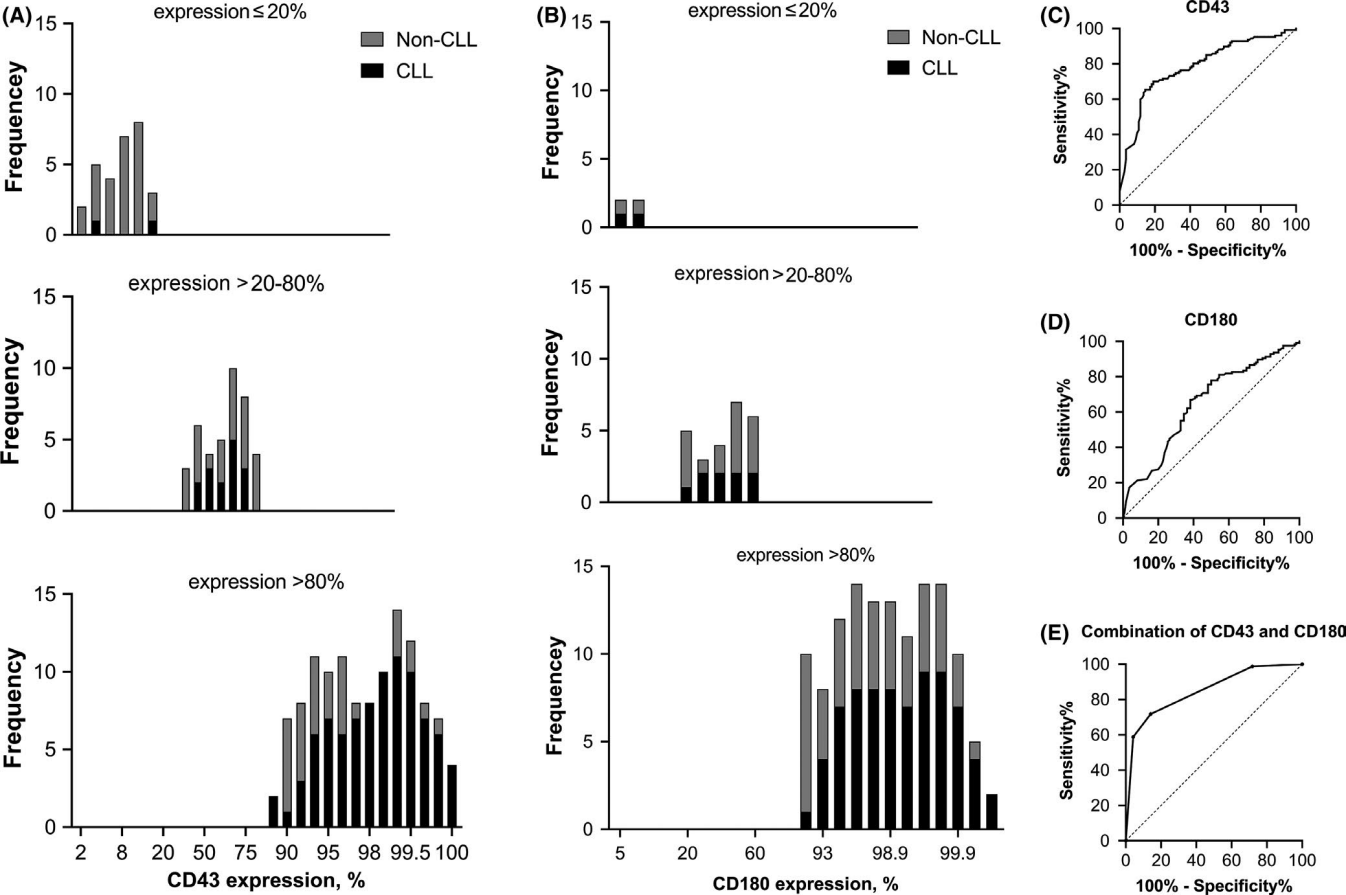
Expression of CD43 and CD180 in CLL cases in exploratory cohort. (A) CD43 expression, CLL cases were predominantly located in the “expression” (>80%) group, while non‐CLL cases were principally located in the “negative” (≤20%) and “part expression” (>20%–80%) group (*p* < 0.001). (B) CD180 expression, CLL cases were mainly located in the “expression” group, while non‐CLL cases were mostly located in the “negative” and “part expression” group (*p* = 0.019). (C) ROC curve analysis of CD43 expression in CLL cases versus non‐CLL cases (sensitivity and specificity were 71.8% and 88.7%, respectively, AUC = 0.83). (D) ROC curve analysis of CD180 expression in CLL cases versus non‐CLL cases (sensitivity and specificity were 71.8% and 54.9%, respectively, AUC = 0.65). (E) ROC curve analysis of combination of CD43 and CD180 in CLL cases versus non‐CLL cases (sensitivity and specificity were 62.4% and 91.5%, respectively, AUC = 0.85)

In the exploratory cohort, CD43 positive expression were found in 82 (96.5%) CLL patients and 44 (62.0%) non‐CLL patients. Cases in exploratory cohort were distributed as “negative” (≤20%), “part expression” (>20%–80%) and “expression” (>80%), and assessed according to the expression of CD43. As showed in Figure 1A of CD43 expression, CLL cases were predominantly located in the “expression” group, while non‐CLL cases were principally located in the “negative” and “part expression” group (*p* < 0.001). ROC curve analysis showed that the sensitivity and specificity of CLL diagnosis could be assigned with a sensitivity of 71.8% and a specificity of 88.7% when CD43 expression was measured by positive rate and the area under the curve (AUC) was 0.83 (Figure 1C).

#### 3.2.2 The CD180 expression between CLL and non‐CLL patients

CD180 expression were positive in 83 (97.6%) CLL patients and 69 (97.2%) non‐CLL patients in exploratory cohort. Cases in exploratory cohort were distributed as “negative”, “part expression” and “expression” and assessed according to the expression of CD180. In Figure 1B, CLL cases were mainly located in the “expression” group, while non‐CLL cases were mostly located in the “negative” and “part expression” group (*p* = 0.019). As displayed in Figure 1D, the sensitivity and specificity of CLL diagnosis could be assigned with a sensitivity of 71.8% and a specificity of 54.9% when CD180 expression was measured by positive rate using ROC curve analysis (AUC = 0.65).

The combination of the positive rate of both CD43 and CD180 expression could increase the specificity of CLL diagnosis to 91.5% with a sensitivity of 62.4% using ROC curve analysis with a larger AUC of 0.85 (Figure 1E).

### 3.3 Development of proposed score system in exploratory cohort

**FIGURE 2:**
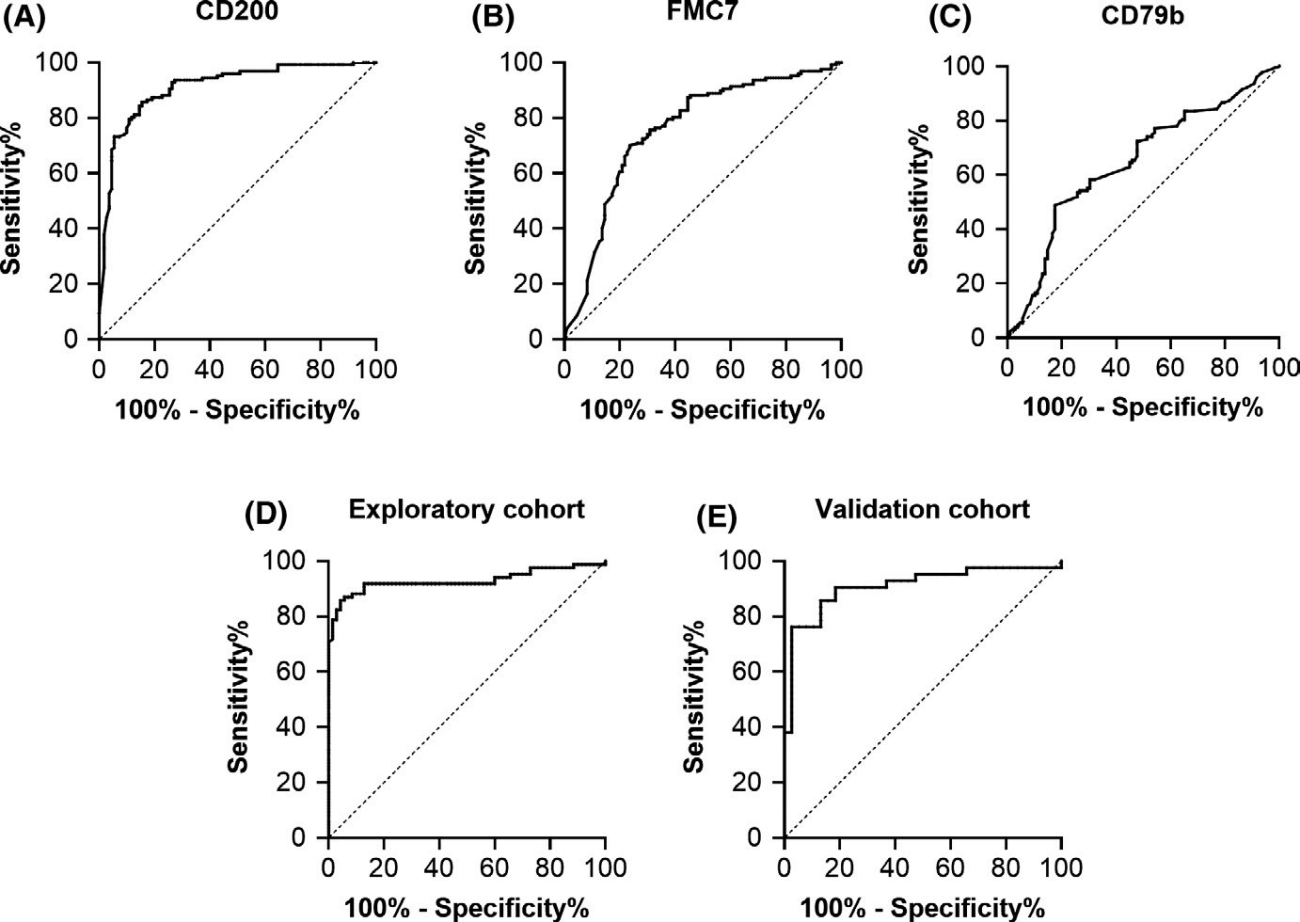
ROC curve of CD200, CD79b, FMC7 expression and combined score of CLL cases vs non‐CLL cases. (A) ROC curve analysis of CD200 expression for CLL cases (sensitivity and specificity were 79.5% and 87.3%, respectively, AUC = 0.89). (B) ROC curve analysis of FMC7 expression for CLL cases (sensitivity and specificity were 70.1% and 70.0%, respectively, AUC = 0.76). (C) ROC curve analysis of CD79b expression for CLL cases (sensitivity and specificity were 48.8% and 79.1%, respectively, AUC = 0.65). (D) ROC curve analysis of combined score (CD43/CD180‐double positive, CD200, FMC7, and CD79b) in exploratory cohort (sensitivity and specificity were 91.8% and 83.1%, respectively, AUC = 0.93). (E) ROC curve analysis of combined score in validation cohort (sensitivity and specificity were 90.5% and 79.5%, respectively, AUC = 0.91)

We first performed the ROC curve analysis of CD200, FMC7, and CD79b (markers in CLLflow score apart from CD5 or CD23) expression for CLL diagnosis in the exploratory cohort. The sensitivity and specificity of CD200 expression in CLL diagnosis was 79.5% and 87.3%, respectively when CD200 expression was measured by positive rate (AUC = 0.89, Figure 2A). As displayed in Figure 2B, the diagnostic value of CLL could be assigned with a sensitivity of 70.1% and a specificity of 70.0% when FMC7 expression was measured by negative expression (AUC = 0.76). The diagnosis of CLL showed a sensitivity of 48.8% and specificity of 79.1% when CD79b expression was measured by negative expression (AUC = 0.65, Figure 2C).

We then analyzed a combination of the four variables: CD43/CD180‐double positive, CD200, CD79b, and FMC7 (entered as positive percentage of B cells) using logistic regression analysis. As presented in Table S5, multivariate analyses demonstrated that all four variables showed significant predicted value (B value) of CLL diagnosis. A preliminary score based on CLLflow score^15^ was developed using results from the multivariate analysis, calculating as follows:

$$\begin{array}{cc} {\text{Score}}^{\text{preliminary}}= & \%\text{CD}43/\text{CD}180^{\text{pos}}\times 0.003+\%\text{CD}200^{\text{pos}}\times 0.007\\ & \;‐\%\text{FMC}7^{\text{pos}}\times 0.003‐\%\text{CD}79b^{\text{pos}}\times 0.001‐0.003 \end{array}$$

In order to allow easy calculation in clinic, this score was simplified as follows:

$$\text{CLL}\;\text{score}=\%\text{CD}43/\text{CD}180^{\text{pos}}+\%\text{CD}200^{\text{pos}}‐\%\text{FMC}7^{\text{pos}}‐\%\text{CD}79b^{\text{pos}}$$

**FIGURE 3:**
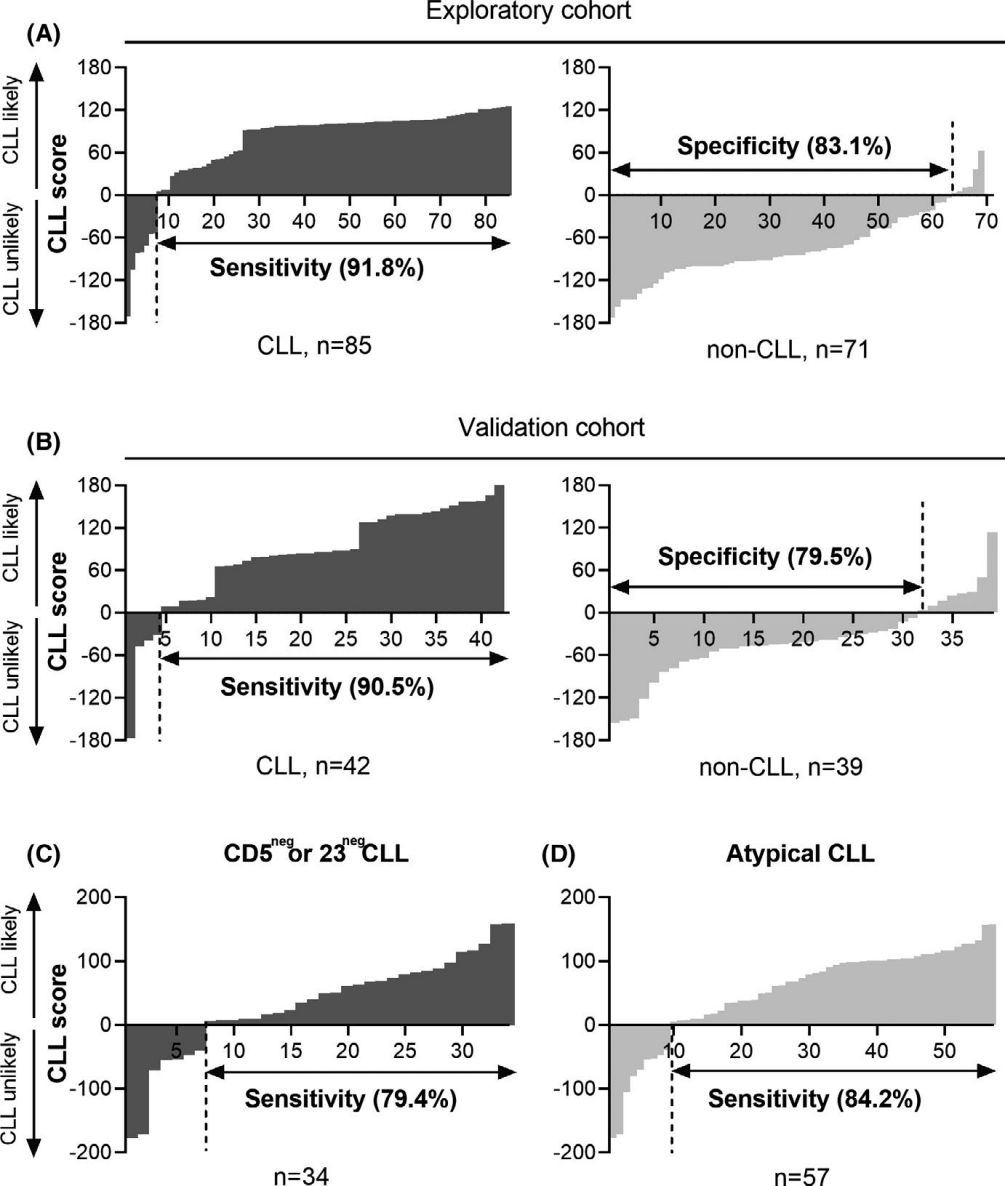
The distribution of combined scoring values of CLL and non‐CLL group. (A) Individual CLL score values for all cases in the exploratory cohort (n = 156). CLL cases showed sensitivity were left, and non‐CLL cases showed specificity were right. (B) Individual CLL score values for all cases in the validation cohort (n = 81). CLL cases showed sensitivity were left, and non‐CLL cases showed specificity were right. (C) Individual CLL score values for all cases in CD5 negative or CD23 negative CLL group showing sensitivity. (D) Individual CLL score values for all cases in atypical CLL group showing sensitivity

A diagnosis of CLL is likely when the CLL score is higher than zero. This score showed a sensitivity of 91.8% and a specificity of 83.1%, with an increased AUC (0.93) compared to each single variable (Figure 2D). The distribution of scoring values in CLL and non‐CLL group were showed in Figure 3A. This CLL score showed comparable sensitivity (91.8% vs. 83.5%, *p* = 0.065) and specificity (83.1% vs. 77.5%, *p* = 0.388) versus the Moreau score, and significantly increased sensitivity (91.8% vs. 83.5%, *p* = 0.039) and comparable specificity (83.1% vs. 91.5%, *p* = 0.070) versus the CLLflow score (Tables 1 and S6).

**TABLE 1:** Comparison of diagnostic value of three score systems in exploratory cohorts

Score	Moreau	CLLflow	Atypical CLL	*P*1 value	*P*2 value
Exploratory cohort					
Sensitivity	83.5%	83.5%	91.8%	0.065	0.039
Specificity	77.5%	91.5%	83.1%	0.388	0.070
Validation cohort					
Sensitivity	81.0%	83.3%	90.5%	0.289	0.250
Specificity	71.8%	87.2%	79.5%	0.508	0.375

Abbreviations: CLL, chronic lymphocytic leukemia. *P*1, compared between Moreau score and Atypical CLL score using McNemar test; *P*2, compared between CLLflow score and Atypical CLL score using McNemar's test.

### 3.4 Confirmation of the proposed score in validation cohort

The proposed CLL score displayed a sensitivity of 90.5% and a specificity of 79.5% for CLL diagnosis in the validation cohort, with an AUC of 0.91 (Figure 2E), which were consistent with the exploratory cohort (*p* = 0.808 of the sensitivity and *p* = 0.639 of the specificity). Above‐mentioned results demonstrated that it is a model with a good level of diagnostic ability. The distribution of scoring values in CLL and non‐CLL group were showed in Figure 3B. The sensitivity and specificity were comparable with previous scores in the validation cohort (Table 1 and S6) (sensitivity 90.5% for proposed score vs. 81.0% for the Moreau score, *p* = 0.289, specificity 79.5% vs. 71.8%, *p* = 0.508; sensitivity 90.5% for proposed score vs. 83.3% for the CLLflow score, *p* = 0.250; specificity 79.5% vs. 87.2%, *p* = 0.375).

The specificity and sensitivity of the new CLL score was calculated with and without CD180, respectively. The sensitivity was the same between with and without CD180, nevertheless, the specificity with CD180 inclusion (59/71, 83.1%; 31/39, 79.5%; AUC = 0.93 and 0.91) was higher than without (56/71, 78.9%; 29/39, 74.4%; AUC = 0.91 and 0.90) in exploratory and validation cohort, respectively.

### 3.5 Improved sensitivity of the proposed score in atypical CLL cases and CD5 negative or CD23 negative CLL cases

**FIGURE 4:**
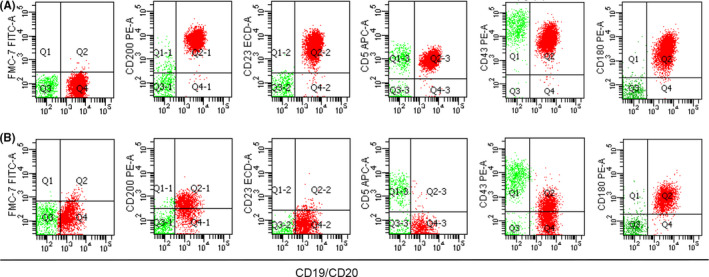
Representative flow cytometry in typical CLL and atypical CLL. (A) The immunophenotype of a typical CLL patient showed positive expression of CD5, CD23, CD200, CD43, and CD180, negative expression of FMC7. (B) The immunophenotype of an atypical CLL patient showed negative expression of CD5, CD23, and FMC7, positive expression of CD200, CD43, and CD180

There were 57 (44.9%) atypical CLL cases and 34 (26.8%) CD5 negative or CD23 negative CLL cases in this study, whereas CD43 negative or CD180 negative CLL cases were only 14 (11.0%). We first performed evaluation on atypical CLL group and CD5 negative or CD23 negative CLL group using Moreau score and CLLflow score. In atypical CLL group, sensitivity of CLL diagnosis was just 61.4% for Moreau score and 64.9% for CLLflow score. In CD5 negative or CD23 negative CLL cases, the sensitivity decreased to 41.2% in Moreau score and 47.1% in CLLflow score. We then used the proposed CLL score to evaluate atypical CLL group and CD5 negative or CD23 negative CLL group. This proposed score, we called atypical CLL score, markedly improved the sensitivity of CLL diagnosis to 84.2% in atypical CLL group and 79.4% in CD5 negative or CD23 negative CLL group, respectively (Table 2). The distribution of scoring values of CD5 negative or CD23 negative CLL is showed in Figure 3C and the distribution of scoring values of atypical CLL is showed in Figure 3D. The representative flow cytometry of typical CLL and atypical CLL is presented in Figure 4.

**TABLE 2:** Diagnostic value of atypical and CD5 negative or CD23 negative CLL cases

Score	Atypical CLL patients (n = 57)	*P* value	CD5 negative or CD23 negative CLL patients (n = 34)	*P* value
Moreau		0.004		0.002
0–2	22 (38.6%)		20 (58.8%)	
3	35 (61.4%)		13 (38.2%)	
4–5	0 (0%)		1 (3.0%)	
CLLflow		0.001		0.006
>0	37 (64.9%)		16 (47.1%)	
≤0	20 (35.1%)		18 (52.9%)	
Atypical CLL				
>0	48 (84.2%)		27 (79.4%)	
≤0	9 (15.8%)		7 (20.6%)	

Abbreviations: CLL, chronic lymphocytic leukemia. *P* value was compared with atypical CLL score using McNemar's test.

## 4 DISCUSSION

CLL is common in European descents but rare in Asians,^31^ with an incidence rate ratio of 20‐fold more in United States than in Japan.^10^ A study reported higher incidence of unusual morphological variants in Japanese CLL patients compared with patients of European descents.^12^ Using Matutes score system, 87% CLL patients in Europe have a score >3.^8^ In contrast, only 44% Koreans with CLL^11^ and only 52% Japanese with CLL^12^ had a such score. In this study with Chinese patients, only 55.3% of CLL cases had a score >3 using Moreau score, far less than the 91.8% in Europe.^9^ Moreover, the current study showed a sensitivity of 83.5% using CLLflow score in China, far less than the 97.1% in Germany.^15^ These differences suggested that Asian patients with CLL may have different immunophenotypic characteristics and may require different diagnostic scoring systems. This atypical CLL score developed in Chinese patients might be a better measure for CLL patients in Asia.

Moreau score has been long applied in CLL diagnosis, nevertheless, it usually requires double positivity of CD5 and CD23 to obtain a typical immunophenotype.^9^, ^20^ Additionally, the definition of “weak” expression on SmIg is highly relied on the assessment of investigators and might not be easily reproducible.^15^ A study reported that a new CLLflow score including combination of CD5/CD23, FMC7, CD79b, and CD200 showed sensitivity and specificity of 97.1% and 87.2%, respectively in the diagnosis of CLL.^15^ However, MCL could be CD23 positive^32^ and for CLL cases with intermediate CD23 positivity (30%‐92.5%) can either belong to CLL or MCL.^33^ In this study, we replaced CD5 and CD23 with CD43 and CD180 and obtained a significantly improved sensitivity of 79.4% for the diagnosis of CD5 negative or CD23 negative CLL. Therefore, 38.2% (79.4%–41.2%) CLL cases falsely calculated as non‐CLL using Moreau score and 32.3% (79.4%–47.1%) CLL cases falsely evaluated as non‐CLL using CLLflow score (Table S7) were accurately identified as CLL according to our atypical CLL score.

CD43, also known as sialophorin, is a heavily glycosylated transmembrane protein and plays a role in apoptosis modulation, differentiation, and immune homeostasis.^34^ CD43 expressed on the surface of T cells and a subset (13%–23%) of normal B cells.^19^ Recently, many studies have reported that the significantly different expression of CD43 in CLL and other mature B‐cell neoplasms could improve the differential diagnosis.^13^, ^19^, ^20^, ^35^ CD180 also had differential expression among mature B‐cell neoplasms and was identified to have a sensitivity of 77% and a specificity of 92% for the diagnosis of MZL.^24^, ^25^, ^26^ In CLL, CD180 could extend the proliferation and survival of B cells,^36^ and was involved in susceptibility to fludarabine.^37^ In this study, the positive percentage of CD43 and CD180 was more sensitive than previous scores (Moreau score and CLLflow score) in the diagnosis of atypical CLL and CD5 negative or CD23 negative CLL. Above‐mentioned results indicated the potential use of atypical CLL score including CD43 and CD180 in CLL, especially in CD5 negative or CD23 negative CLL cases.

There was no difference in the expression levels of CD43 between atypical and typical CLL cases.^35^ Moreover, CD43 expression was positive in 95.7% of atypical CLL cases, while in MCL, its expression was positive in 39.4% of cases.^38^ Above evidences suggested that CD43 was useful in recognizing atypical CLL from MCL cases. The positive rate of CD180 in MZL and hairy cell leukemia (HCL) were markedly higher than in CLL,^25^ which could help to identify CLL cases from MZL and HCL cases. Therefore, the combination of CD43 and CD180 in this new CLL score could improve the diagnostic value for CLL.

Atypical CLL is predominantly confused with MCL which expresses CD5, sometimes together with CD23 similar to CLL.^14^, ^32^, ^33^ Negative expression of CD5 or CD23 frequently occurred in atypical CLL.^13^ CD43 showed consistent expression in both atypical and typical CLL, and the positive rate of CD43 was higher in CLL compared to that in MCL.^13^, ^35^ CD43 had a relatively high sensitivity up to 100%; however, the specificity of CD43 alone was not sufficient for accurate diagnosis.^20^ Due to the relatively low specificity of CD43,^20^ CD180 was used together with CD43 to improve the specificity in the diagnosis of CLL. CD200 is described as positive in CLL but negative in MCL.^39^ More importantly, CD200 is particularly useful in recognizing atypical CLL.^14^ The AUC (0.89) of CD200 is the highest among the five markers (CD43, CD180, CD200, CD79b, and FMC7) used in this new CLL score. Therefore, CD200 is a more specific marker to diagnose CLL as compared to CD43 and CD180. As CD43 is also expressed among MCL, the current scoring system might have been successful due to the inclusion of CD200. Generally, the CD79b and FMC7 tend to show negative expression in CLL than MCL, and CD79b and FMC7 are useful markers for the differential diagnosis of CLL.^15^ In this study, the diagnostic value of a combination of CD43/CD180, CD200, FMC7, and CD79b was increased than that of each single marker. Furthermore, the application of atypical CLL score in the diagnosis of CLL required further confirmation in large cohort.

To the best of our knowledge, this study demonstrated the diagnostic value of a combination of CD43 and CD180 with CD200, FMC7, and CD79b in CD5 negative or CD23 negative CLL for the first time. Replacement of CD5 and CD23 with CD43 and CD180 in CLLflow score showed comparable sensitivity and specificity with Moreau score and CLLflow score in CLL diagnosis. Moreover, this replacement significantly improved sensitivity in the diagnosis of CD5 or CD23 negative CLL and atypical CLL. Therefore, double positive for CD43 and CD180, together with other B‐cell surface markers could improve the sensitivity of the diagnosis in CLL patients with negative expression of CD5 or CD23.

Moreau score and CLLflow score are demonstrated to have well diagnostic value for typical CLL cases.^8^, ^9^, ^15^ In contrast, the atypical CLL score proposed in this study is of great significance for atypical CLL and CD5 negative or CD23 negative CLL. Taken together, this atypical CLL score predominately serves as a complementary algorithm for improving diagnostic value for atypical CLL and CD5 negative or CD23 negative CLL cases. Mature B‐cell neoplasms in Asia and undifferentiated cases of European descents with a Moreau score ≤3, as well as with a CLL flow score ≤0, are fitted for assessment with this atypical CLL score.

## 5 CONCLUSIONS

CD43 and CD180 showed a relatively high expression on CLL, as well as in atypical CLL and CD5 negative or CD23 negative CLL. Replacement of CD5 and CD23 in previous diagnostic score of CLL (Moreau score and CLLflow score) could obtain comparable sensitivity and specificity in the diagnosis of CLL. This atypical CLL score proposed in this study helped to offer an accurate differentiation of CLL from non‐CLL malignancies, particularly in CD5 or CD23‐negative mature B‐cell neoplasms with a clinical suspicion of CLL.

## CONFLICT OF INTEREST

The authors declare no conflict of interest.

## AUTHOR CONTRIBUTIONS

YL, XM, and DZ presided over the overall research idea of this study, participated in or supervised literature retrieval, research design, data collection, data analysis, and wrote manuscripts. XT, LH, LL, CW, CH, SL, ZW, and MX conduct data collection and analysis. All the authors approved the final proof.

## ETHICAL APPROVAL

The present study was approved by the Ethical Committee of Tongji Hospital, Tongji Medical College, Huazhong University of Science and Technology (TJ‐IRB20200716), and all procedures conducted were in agreement with the Declaration of Helsinki.

## Supporting information

Fig S1

Table S1

Table S2

Table S3

Table S4

Table S5

Table S6

Table S7

## Data Availability

All data are available in the manuscript.
