# Supplementary figures and images for: A new score including CD43 and CD180: Increased diagnostic value for atypical chronic lymphocytic leukemia
Source: Cancer Med. 2021 Jun 1;10(13):4387–96. doi: 10.1002/cam4.3983 (PMC8267114; doi:10.1002/cam4.3983)

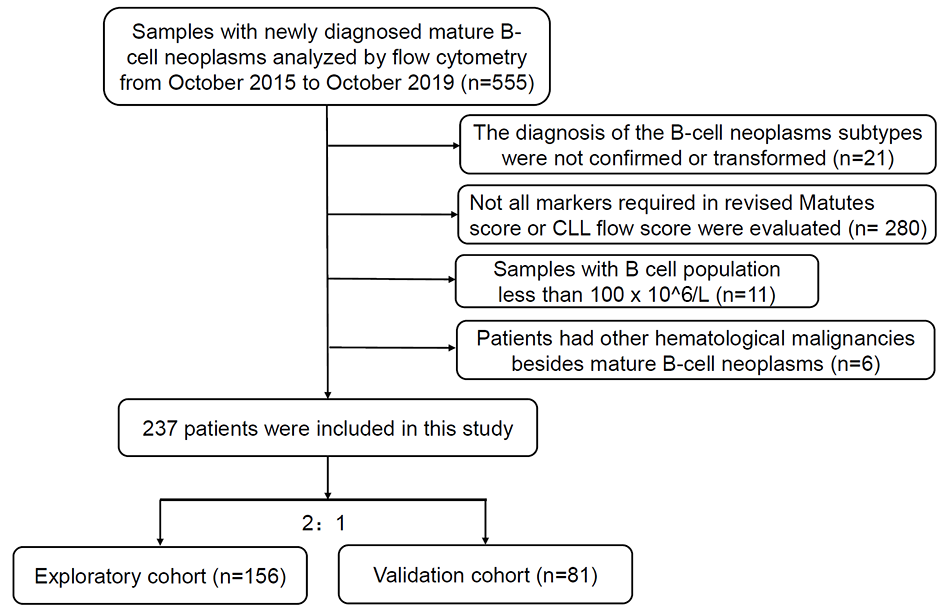

Supplement: Supplementary file 1 — Fig S1 [file CAM4-10-4387-s008.tif]
