# Supplementary material for: A new score including CD43 and CD180: Increased diagnostic value for atypical chronic lymphocytic leukemia
Source: Cancer Med. 2021 Jun 1;10(13):4387–96. doi: 10.1002/cam4.3983 (PMC8267114; doi:10.1002/cam4.3983)
Supplement: Supplementary file 2 — Table S1 [file CAM4-10-4387-s007.doc]

**TABLE S1** Fluorochromes, clones and sources of staining antibodies used.

| Antigen | Fluorochrome | Clone | Source |
| --- | --- | --- | --- |
| CD5 | APC | 652840 | Becton Dickinson |
| CD19 | APC | HIB19 | Becton Dickinson |
| CD20 | APC-Cy7 | L27 | Becton Dickinson |
| CD23 | ECD | IM3609U | Beckman Coulter |
| CD43 | PE | 1G10 | Becton Dickinson |
| CD79b | PE | CB3-1 | Biolegend |
| CD180 | PE | MHR73-11 | Biolegend |
| CD200 | PE | 552475 | Becton Dickinson |
| FMC7 | FITC | 340919 | Becton Dickinson |
| kappa | FITC | FR481 | Dako |
| lambda | PE | FR481 | Dako |

APC: Allophycocyanin; APC-Cy7: Allophycocyanin-Cyanine-7; ECD: Phycoerythrin-Texas Red-x; FITC: Fluorescein Isothiocyanate; PE: Phycoerythrin.
