# Supplementary material for: A new score including CD43 and CD180: Increased diagnostic value for atypical chronic lymphocytic leukemia
Source: Cancer Med. 2021 Jun 1;10(13):4387–96. doi: 10.1002/cam4.3983 (PMC8267114; doi:10.1002/cam4.3983)
Supplement: Supplementary file 3 — Table S2 [file CAM4-10-4387-s004.doc]

**Table S2** Diagnosis of patients in exploratory and validation cohorts.

| characteristics | All patients (N=237) | Exploratory cohort (n=156) | Validation cohort (n=81) |
| --- | --- | --- | --- |
| CLL | 127 (53.6%) | 85 (54.5%) | 42 (51.9%) |
| DLBCL | 15 (6.3%) | 10 (6.4%) | 5 (6.2%) |
| Burkitt | 3 (1.3%) | 2 (1.3%) | 1 (1.2%) |
| MCL | 28 (11.8%) | 19 (12.2%) | 9 (11.1%) |
| FL | 32 (13.5%) | 20 (12.8%) | 12 (14.8%) |
| MZL | 17 (7.2%) | 10 (6.4%) | 7 (8.6%) |
| MALT | 3 (1.3%) | 2 (1.3%) | 1 (1.2%) |
| LPL | 12 (5.1%) | 8 (5.1%) | 4 (4.9%) |

CLL, chronic lymphocytic leukemia; DLBCL, diffuse large B-cell lymphoma; MCL, mantle cell lymphoma; FL, follicular lymphoma; MZL, marginal-zone lymphoma; MALT, extranodal marginal-zone lymphoma of mucosa-associated lymphoid tissue; LPL, lymphoplasmacytic lymphoma.
