# Supplementary material for: A new score including CD43 and CD180: Increased diagnostic value for atypical chronic lymphocytic leukemia
Source: Cancer Med. 2021 Jun 1;10(13):4387–96. doi: 10.1002/cam4.3983 (PMC8267114; doi:10.1002/cam4.3983)
Supplement: Supplementary file 4 — Table S3 [file CAM4-10-4387-s002.doc]

**Table S3** Baseline characteristics of CLL patients in exploratory and validation cohorts.

| Characteristics | All patients (N=127) | Exploratory cohort (n=85) | Validation cohort (n=42) | *P* value |
| --- | --- | --- | --- | --- |
| Age, median (range) | 61 (35-84) | 60 (35-81) | 62 (40-84) | 0.063 |
| Sex |  |  |  | 0.376 |
| Male | 84 (66.9%) | 54 (63.5%) | 30 (71.4%) |  |
| Female | 43 (33.1%) | 31 (36.5%) | 12 (28.6%) |  |
| Binet stage |  |  |  | 0.831 |
| A | 39 (30.7%) | 26 (30.6%) | 13 (31.0%) |  |
| B | 44 (34.6%) | 30 (35.3%) | 14 (33.3%) |  |
| C | 44 (34.6%) | 29 (34.1%) | 15 (35.7%)) |  |
| Rai stage |  |  |  | 0.884 |
| 0 | 7 (5.5%) | 5 (5.9%) | 2 (4.8%) |  |
| I | 33 (26.0%) | 23 (27.1%) | 10 (23.8%) |  |
| II | 31 (24.4%) | 22 (25.9%) | 9 (21.4%) |  |
| III | 21 (16.5%) | 14 (16.5%) | 7 (16.7%) |  |
| IV | 35 (27.6%) | 21 (24.7%) | 14 (33.3%) |  |
| IPI score |  |  |  | 0.865 |
| Low | 33 (26.0%) | 23 (27.1%) | 10 (23.8%) |  |
| Intermediate | 30 (23.6%) | 21 (24.7%) | 9 (21.4%) |  |
| High | 42 (33.1%) | 26 (30.6%) | 16 (38.1%) |  |
| Very high | 22 (17.3%) | 15 (17.6%) | 7 (16.7%) |  |
| Sample |  |  |  | 0.364 |
| Bone marrow | 98 (77.2%) | 69 (81.2%) | 29 (69.0%) |  |
| Lymph node* | 11 (8.6%) | 6 (7.0%) | 5 (12.0%) |  |
| Peripheral blood | 18 (14.2%) | 10 (11.8%) | 8 (19.0%) |  |
| Gene mutation |  |  |  | 0.444 |
| IGHV | 40 (31.5%) | 27 (31.8%) | 13 (31.0%) |  |
| P53 | 12 (9.4%) | 8 (9.4%) | 4 (9.5%) |  |
| MyD88 | 2 (1.6%) | 2 (2.4%) | 0 (0%) |  |
| Untested | 41 (32.3%) | 25 (29.4%) | 16 (38.1%) |  |
| Abnormal chromosome |  |  |  | 0.933 |
| 11q- | 4 (3.1%) | 3 (3.5%) | 1 (2.4%) |  |
| +12 | 18 (14.2%) | 12 14.1(%) | 6 (14.3%) |  |
| 13q- | 37 (29.1%) | 26 (30.6%) | 11 (26.2%) |  |
| 17p- | 7 (5.5%) | 5 (5.9%) | 2 (4.8%) |  |
| Untested | 42 (33.1%) | 26 (30.6%) | 16 (38.1%) |  |

CLL, chronic lymphocytic leukemia; IPI, international prognostic index. * 11 CLL patients underwent flow cytometric tests for samples from both lymph nodes and bone marrow or peripheral blood.
