# Supplementary material for: A new score including CD43 and CD180: Increased diagnostic value for atypical chronic lymphocytic leukemia
Source: Cancer Med. 2021 Jun 1;10(13):4387–96. doi: 10.1002/cam4.3983 (PMC8267114; doi:10.1002/cam4.3983)
Supplement: Supplementary file 5 — Table S4 [file CAM4-10-4387-s005.docx]

**Table S4** Comparison of characteristics of CLL patients with CD5 positivity and negativity.

| Characteristics | CD5 positive (n=115) | CD5 negative (n=12) | *P* value |
| --- | --- | --- | --- |
| Age, median (range) | 60 (35-81) | 62 (49-73) | 0.089 |
| Sex |  |  | 0.548 |
| Male | 77 (67.0%) | 7 (71.4%) |  |
| Female | 38 (33.0%) | 5 (28.6%) |  |
| Binet stage |  |  | 0.845 |
| A | 36 (31.3%) | 3 (25.0%) |  |
| B | 40 (34.8%) | 4 (33.3%) |  |
| C | 39 (33.9%) | 5 (41.7%)) |  |
| Rai stage |  |  | 0.752 |
| 0 | 7 (6.1%) | 0 (0%) |  |
| I | 31 (27.0%) | 2 (16.7%) |  |
| II | 28 (24.3%) | 3 (25.0%) |  |
| III | 18 (15.7%) | 3 (25.0%) |  |
| IV | 31 (27.0%) | 4 (33.3%) |  |
| IPI score |  |  | 0.998 |
| Low | 30 (26.1%) | 3 (25.0%) |  |
| Intermediate | 27 (23.5%) | 3 (25.0%) |  |
| High | 38 (33.0%) | 4 (33.3%) |  |
| Very high | 20 (17.4%) | 2 (16.7%) |  |
| Sample |  |  | 0.541 |
| Bone marrow | 90 (78.3%) | 8 (66.7%) |  |
| Lymph node* | 9 (7.8%) | 2 (16.7%) |  |
| Peripheral blood | 16 (13.9%) | 2 (16.7%) |  |
| Gene mutation |  |  | 0.128 |
| IGHV | 37 (48.1%) | 3 (33.0%) |  |
| P53 | 11 (14.3%) | 1 (11.1%) |  |
| MyD88 | 1 (1.3%) | 1 (11.1%) |  |
| Untested | 38 (33.0%) | 3 (25.0%) |  |
| Abnormal chromosome |  |  | 0.866 |
| 11q- | 3 (3.9%) | 1 (11.1%) |  |
| +12 | 16 (21.1%) | 2 (22.2%) |  |
| 13q- | 33 (43.4%) | 4 (44.4%) |  |
| 17p- | 6 (7.9%) | 1 (11.1%) |  |
| Untested | 39 (33.9%) | 3 (25.0%) |  |

CLL, chronic lymphocytic leukemia; IPI, international prognostic index. * 11 CLL patients underwent flow cytometric tests for samples from both lymph nodes and bone marrow or peripheral blood.
