# Supplementary material for: A new score including CD43 and CD180: Increased diagnostic value for atypical chronic lymphocytic leukemia
Source: Cancer Med. 2021 Jun 1;10(13):4387–96. doi: 10.1002/cam4.3983 (PMC8267114; doi:10.1002/cam4.3983)
Supplement: Supplementary file 6 — Table S5 [file CAM4-10-4387-s006.doc]

**Table S5** Logistic regression of variables in the score system.

| Variable | B | Standard error | 95% CI of B | *P* value |
| --- | --- | --- | --- | --- |
| CD43/CD180 | 0.003 | 0.060 | 0.001-0 .004 | <0.001 |
| CD200 | 0.007 | 0.001 | 0.006-0.008 | <0.001 |
| CD79b | -0.001 | <0.001 | -0.001-0 | 0.019 |
| FMC7 | -0.003 | 0.001 | -0.004- (-0.001) | 0.001 |
| Constant | 0.003 | 0.001 | -0.116-0.123 | 0.955 |

CLL, chronic lymphocytic leukemia; CI, confidence interval.
