# Supplementary material for: A new score including CD43 and CD180: Increased diagnostic value for atypical chronic lymphocytic leukemia
Source: Cancer Med. 2021 Jun 1;10(13):4387–96. doi: 10.1002/cam4.3983 (PMC8267114; doi:10.1002/cam4.3983)
Supplement: Supplementary file 7 — Table S6 [file CAM4-10-4387-s003.doc]

**Table S6** Comparison of diagnostic value of three score systems in exploratory and validation cohorts.

| Score | Exploratory cohort | | Validation cohort | |
| --- | --- | --- | --- | --- |
|  | CLL patients (n=85) | Non-CLL patients (n=71) | CLL patients (n=42) | Non-CLL patients (n=39) |
| Moreau |  |  |  |  |
| 0-2 | 14 (16.5%) | 55 (77.5%) | 8 (19.0%) | 28 (71.8%) |
| 3 | 24 (28.2%) | 15 (21.1%) | 11 (26.2%) | 10 (25.6%) |
| 4-5 | 47 (55.3%) | 1 (1.4%) | 23 (54.8%) | 1 (2.6%) |
| CLLflow |  |  |  |  |
| >0 | 71 (83.5%) | 6 (8.5%) | 35 (83.3%) | 5 (12.8%) |
| ≤0 | 14 (16.5%) | 65 (91.5%) | 7 (16.7%) | 34 (87.2%) |
| Atypical CLL* |  |  |  |  |
| >0 | 78 (91.8%) | 12 (16.9%) | 38 (90.5%) | 8 (20.5%) |
| ≤0 | 7 (8.2%) | 59 (83.1%) | 4 (9.5%) | 31 (79.5%) |

CLL, chronic lymphocytic leukemia. **P* = 0.808 of the sensitivity and *P* = 0.639 of the specificity compared between exploratory cohort and validation cohort using χ2 test.
