# Supplementary material for: A new score including CD43 and CD180: Increased diagnostic value for atypical chronic lymphocytic leukemia
Source: Cancer Med. 2021 Jun 1;10(13):4387–96. doi: 10.1002/cam4.3983 (PMC8267114; doi:10.1002/cam4.3983)
Supplement: Supplementary file 8 — Table S7 [file CAM4-10-4387-s001.doc]

**Table S7** Positive rate of antigen expression of atypical CLL falsely evaluated as non-CLL using CLLflow score.

| Cases | CD5 | CD23 | CD43 | CD180 | CD200 | CD79b | FMC7 | CLLflow score | Atypical CLL score |
| --- | --- | --- | --- | --- | --- | --- | --- | --- | --- |
| 1 | 2% | 2% | 74% | 100% | 99% | 95% | 18% | -12 | 60 |
| 2 | 2% | 3% | 100% | 43% | 99% | 98% | 13% | -10 | 31 |
| 3 | 3% | 5% | 65% | 95% | 98% | 99% | 9% | -7 | 55 |
| 4 | 5% | 20% | 98% | 94% | 95% | 99% | 7% | -6 | 83 |
| 5 | 8% | 18% | 94% | 88% | 100% | 92% | 19% | -3 | 77 |
| 6 | 15% | 37% | 90% | 93% | 90% | 90% | 45% | -30 | 45 |
| 7 | 16% | 55% | 98% | 92% | 95% | 91% | 37% | -17 | 59 |
| 8 | 18% | 86% | 96% | 95% | 92% | 68% | 55% | -13 | 64 |
| 9 | 20% | 62% | 99% | 98% | 80% | 49% | 72% | -21 | 57 |
| 10 | 2% | 3% | 50% | 98% | 65% | 90% | 10% | -33 | 15 |
| 11 | 92% | 35% | 95% | 99% | 2% | 70% | 25% | -58 | 2 |

CLL, chronic lymphocytic leukemia.
